# Deferasirox-induced hyperammonemia and Fanconi syndrome: a case report

**DOI:** 10.3389/fped.2024.1461867

**Published:** 2024-10-10

**Authors:** Houfu Zhou, Daoxue Xiong, Yan Feng, Jianyu Jiang

**Affiliations:** Intensive Care Unit, Chongqing University Three Gorges Hospital, Chongqing, Wanzhou, China

**Keywords:** hyperchloremic acidosis, deferasirox hyperchloremic, metabolic acidosis, hyperammonemia, Fanconi syndrome

## Abstract

**Background:**

The exact mechanism of hyperammonemia is thought to be multifactorial, but is not yet fully understood. No studies have yet reported hyperammonemia combined with Fanconi syndrome caused by deferasirox.

**Case presentation:**

A 10-year-old girl was admitted for vomiting and altered consciousness. Blood testing revealed hyperammonemia and normal liver and coagulation functions. During hospitalization, the patient also exhibited hyperchloremic metabolic acidosis, hypokalemia, hyponatremia, and hypophosphatemia. Additionally, urinalysis revealed glucose and protein levels clinically consistent with Fanconi syndrome. The patient had a history of severe beta-thalassemia and had received intermittent blood transfusions for approximately ten years. The patient had been administered oral deferasirox at a 400 mg/day dose at the age of four, which had been gradually increased to the current 750 mg/day dosage. Upon admission, deferasirox was discontinued and treatment including mechanical ventilation, continuous blood purification therapy for ammonia reduction and acidosis, and electrolyte imbalance corrections was administered. Subsequently, serological markers returned to normal, urine test findings improved. To the best of our knowledge, this is the first report of a case of hyperammonemia with Fanconi syndrome owing to deferasirox.

**Conclusions:**

For effective management and long-term follow-up of chronic diseases in children, pediatricians must master standardized treatments and the adverse reactions of various drugs. When symptoms are difficult to explain clinically, we must trace the source and adjust the treatment plan to maximize improving the patient's prognosis.

## Background

Often fatal, hyperammonemia is a clinical disease that can manifest as altered consciousness. The condition is mainly observed in children with congenital metabolic urea cycle (UC) abnormalities, hepatic encephalopathy, UC disorders, and organic acidemia; however, the exact mechanism is not fully understood and is considered multifactorial (1, 2). Fanconi syndrome is a defect in the proximal renal tubules that leads to poor absorption of certain electrolytes and substances that are typically absorbed by the proximal tubules. This defect results in low-molecular-weight proteinuria, hypokalemia, hypophosphatemia, metabolic acidosis, hypouricemia, and renal glycosuria, and may be associated with medication (3). Herein, we report the first case of hyperammonemia combined with Fanconi syndrome caused by deferasirox (DFR).

## Case presentation

A 10-year-old girl was admitted to the Pediatric Intensive Care Medicine Department at Chongqing University Three Gorges Hospital because of a 1-day history of persistent vomiting and a disturbance in consciousness that had lasted one half-day. The day preceding admission, the patient presented with frequent nonpropulsive vomiting without obvious induction. No other discomforts such as abdominal pain, headache, dizziness, convulsions, or cough were present. Half a day before admission, the patient had suddenly presented with consciousness disturbance and dyspnea without external stimuli response or cyanosis. An arterial blood gas analysis at the local hospital indicated metabolic acidosis [pH: 7.235, lactate: 3.2 mmol/L, base excess (BE): −16.5 mmol/L]. The patient was transferred to our hospital after the blood ammonia concentration exceeded 500 µmol/L. While there was no past medical history of abnormalities at birth, the patient had been diagnosed with β-thalassemia (severe) at four months and had subsequently undergone intermittent long-term blood transfusions of 1–2 donor units every 3–4 weeks. Additionally, at the age of four, oral DFR (Novartis Pharma Stein AG) treatment was initiated at 400–500 mg/day for iron removal. At treatment initiation, the patient’s ferritin levels consistently exceeded 1,200 ng/ml. When the dosage was gradually increased to 750 mg/day, at which it remained for nearly one year, a downward trend in ferritin levels was seen. The ferritin level upon admission was 800 ng/ml. Vital sign evaluation revealed a 36.8°C body temperature, 124/86 mmHg blood pressure, 122 beats/min heart rate, 20 breaths/min respiratory rate, 88% transcutaneous oxygen saturation (under 2 L oxygen via a face mask), and 22 kg weight. Regarding the comatose state of the patient, their Glasgow Coma Scale score was 5 (E1V3M1), with extremely poor response, sniffing-like breathing, irregular rhythm, an inspiratory tricuspid sign, and pale complexion.

An arterial blood gas analysis revealed a 7.33 pH, 74 mmHg PaO_2_, 18 mmHg PaCO_2_, 3.3 mmol/L lactate, 9.5 mmol/L HCO_3_, and −16.4 mmol/L BE. Notable laboratory tests of the patient included a 121 g/L hemoglobin level, 4.8 × 10^9^/L leukocyte count, 231 × 10^9^/L platelet count, 4.14 × 10^12^/L red blood cell count, 61.3% neutrophilic granulocyte ratio, and 31.4% lymphocyte ratio. Routine urine testing revealed 3+ glucose, 1+ urine protein, >150.00 mg/dl urine microalbumin, and 3+ occult blood. Microscopic red blood cell examination revealed 2–4 red blood cells/high power field. Additional notable blood tests included ammonia levels of 584.6 µmol/L (11–51 µmol/L), ferritin levels of 800 ng/ml and renal function: 3.38 mmol/L potassium, 131.0 mmol/L sodium, 111.5 mmol/L chloride, 2.37 mmol/L calcium, 5.3 mmol/L urea, and 47 µmol/L creatinine (Cr). Normal liver and coagulation function was noted. Therefore, admission diagnoses included: (1) hyperammonemia, (2) respiratory failure, (3) metabolic acidosis, (4) electrolyte disturbances, and (5) genetic metabolic diseases?

**Figure 1:**
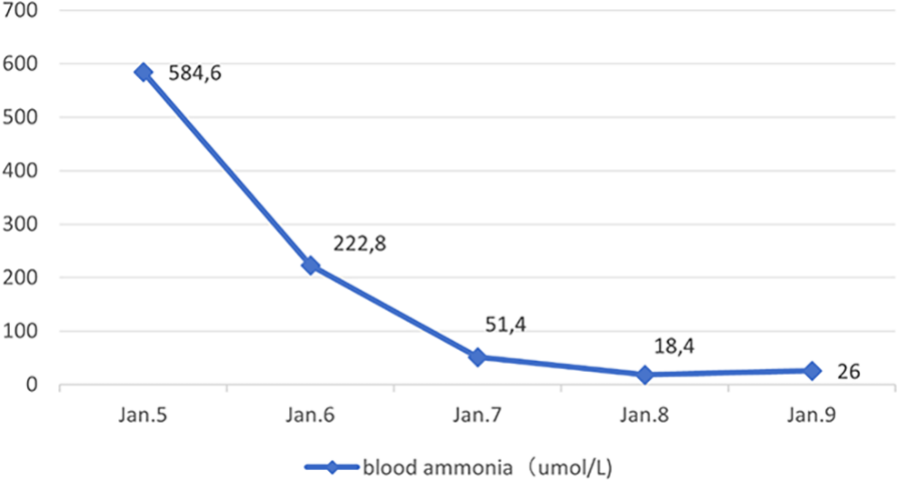
Blood ammonia changes.

Following admission, the patient received mechanical ventilation, mannitol to reduce intracranial pressure, Intravenous injection of 3.0 g of l-arginine hydrochloride was administered within 90 min, followed by intravenous maintenance combined with l-carnitine (1 g i.v. q.d.) to promote nitrogen excretion, in addition to supplementation with vitamins B6, B12, and C. Additionally, the patient was treated with a high-carbohydrate diet. Continuous venovenous hemodiafiltration (CVVHDF) was administered on the day of admission and the following day, for a total duration of 25 h. DFR was stopped during hospitalization. Blood ammonia levels decreased gradually (Figure 1), and the patient was removed from the ventilator once consciousness improved two days later.

However, multiple blood gas and electrolyte tests performed after admission revealed hyperchloremic acidosis, hypokalemia, hyponatremia (Table 1), and multiple abnormal urine test results (Table 2).

**Table 1:** Continuous intra-arterial blood gas changes (*N*: 7.32–7.42).

Date	PH	K (mmol/L)	Na (mmol/L)	Cl (mmol/L)	Ca (mmol/L)	Lac (mmol/L)	CO_2_ (mmHg)	HCO_2_-(mmol/L)	BE (mmol/L)
Jan 5th	7.33	4	139	116.4	1.97	3.3	18	9.5	−16.4
Jan 6th	7.29	3.2	132	111.5	2.37	0.6	21	10.1	−16.5
Jan 7th	7.26	3	127	113.2	1.93	0.3	24	10.8	−16.3
Jan 8th	7.36	2.7	127	109.3	2.04	0.3	26	14.7	−10.7
Jan 9th	7.44	2.9	128	105.6	2.15	1.7	28	19	−5.2
Jan 10th	7.44	2.9	130	98.7	2.11	2.1	32	21.7	−2.2
Jan 13th	7.42	3.8	136	102	2.11	0.4	33	22	−2.3

**Table 2:** Changes in urine routine.

Date	Glu	Protein	Ketone body	Occult blood	Erythrocyte (/HP)
Jan 5th	3+	1+	2+	3+	2–4
Jan 6th	4+	3+	2+	1+	1–3
Jan 7th	4+	3+	1+	1+	1–2
Jan 9th	–	–	1+	–	–
Jan 10th	3+	–	–	–	–
Jan 13th	–	–	–	–	–

Therefore, other relevant auxiliary examinations were conducted: serum beta-hydroxybutyrate: 1,050.00 µmol/L (0–280 µmol/L), inorganic phosphorus: 0.30 mmol/L (1.25–1.93 mmol/L), uric acid: 46 µmol/L (119–330 µmol/L), ferritin: 946.30 ng/ml (<400 ng/ml), and 24-h quantitative urine protein: 2,199.36 mg/24 h (0–120 mg/24 h). Additionally, urine tests were used to assess for early renal tubular lesions: Cr: 2786 µmol/L (2,550–20000 µmol/L), alpha-1-microglobulin: 209.6 mg/g Cr (0–14 mg/g Cr), and N-acetylglucosaminidase: 59.1 U/g Cr (3–18 mg/g Cr). Head magnetic resonance imaging revealed well-defined and symmetrical abnormalities in the thalamus bilaterally with an uncertain nature. Hyperaminomic encephalopathy considered. There were no abnormalities in the amino acid and acylcarnitine spectrum analysis for blood genetic metabolic diseases. Comprehensive urinary analysis of organic acids revealed an increase in 3-hydroxybutyric acid. Additionally, the Trios whole-exome gene test found two heterozygous *HBB* gene mutations, namely *c.126_129del* (*p.Phe42Leufs Ter19*; derived from the father) and *c.52A>T* (*p.Lys18Ter*; derived from the mother), identified as compound heterozygous mutations. Clinical features were consistent with Fanconi syndrome when combined with the presence of hyperchloremic metabolic acidosis, hypokalemia, hyponatremia, hypophosphatemia, hypouricemia, other manifestations, and abnormal urine tests. The child was considered to have hyperammonemia caused by high doses of long-term oral DFR administration, which was accompanied by Fanconi syndrome; therefore, DFR was then discontinued. Sodium bicarbonate was used to correct the acidosis, potassium chloride was used as a potassium supplement, 3% sodium chloride was used to correct hyponatremia, and fructose diphosphate sodium was used to supplement phosphorus. One week after admission, the acidosis and electrolyte imbalance were corrected. However, a routine blood examination revealed a 93 g/L hemoglobin level on January 8th. Subsequently, 2U of red blood cell suspension was infused to correct the anemia on January 10th; two days later, the child was transferred to a general ward. On January 13th, routine urine examination, 24-h urine protein quantification, and early renal tubular damage markers had returned to normal. The patient was discharged from our hospital on January 15th with diagnoses of hyperammonemia, respiratory failure, Fanconi syndrome, and beta-thalassemia. The patient's current condition is attributable to drug side effects, and the thalassemia necessitates continued iron chelation therapy. Moving forward, specialist input for adjusting the medication regimen and dosage will be needed to best minimize these side effects.

Informed consent was obtained from the patient and her guardians for the publication of this case report.

## Discussion and conclusions

This study reported a rare pediatric case of hyperammonemia with Fanconi syndrome in children refers to excessive ammonia accumulation in the blood, which can cause severe consequences, including cerebral edema, severe neurological impairment, and death (4). In this case, the patient presented with vomiting as well as consciousness disorders and was admitted to the hospital in a comatose state with respiratory failure. Blood tests revealed an increased ammonia level of 584.6 µmol/L, meeting the criteria for hyperammonemia. In infants and children, common causes of hyperammonemia include UC disorders or organic acidemia, which can cause increased blood ammonia levels by competitively inhibiting N-acetylglutamate synthase. Moreover, hyperammonemia can have drug-related and miscellaneous causes, including valproate sodium and blood transfusions (5, 6). The patient in this example developed hyperammonemia at a later age; the blood ammonia levels soon returned to normal after aggressive intravenous treatments using arginine and l-carnitine to enhance nitrogen excretion, coupled with CVVHDF. Furthermore, this patient had normal amino acid and acylcarnitine profiles in blood and urine organic acid analyses. No amino acid gene mutations were found in the Trios whole-exome gene test, suggesting that hyperammonemia is highly unlikely to be related to congenital urea and organic acid metabolism disorders. Upon further investigation, the patient was diagnosed with beta-thalassemia (severe type) at four months due to anemia at a local hospital. Subsequently, the patient received long-term treatment with intermittent blood transfusions and began DFR therapy at four years old. The dose was increased from 400 mg/day to the current dose of 750 mg/day, during which ferritin levels fluctuated between 950 ng/ml and 2000 ng/ml. The oral dose was 750 mg/day for nearly a year. Therefore, we believe this hyperammonemia case may have been related to long-term DFR administration. Previous studies have reported that DFR can cause hyperammonemia in children, possibly through intramitochondrial bicarbonate depletion or disruption of other pivotal UC molecules. Additionally, mitochondrial toxicity may cause hepatocyte dysfunction and hyperammonemia in predisposed or vulnerable patients, including pediatric patients (7). Defective alleles of *UGT1A1*, *ABCC2*, and *ABCG2* may contribute to drug-related adverse events (8).

However, according to literature reports, DFR can cause Fanconi syndrome, which is a group of symptoms caused by functional abnormalities of the proximal renal tubules. These abnormalities manifest as renal glycosuria, renal sodium loss, acidosis, tubular proteinuria, hypophosphatemia, and others (3). In the present case, multiple urine tests were positive for glucose, proteins, and occult blood. We found that the patient had no signs of edema or ascites with no decrease in serum albumin when we searched for proteinuria and hematuria causes. Blood lipid levels were normal, while microalbuminuria was increased in routine urine tests. Early renal tubular injury markers revealed that α1-microglobulin and N-acetylglucosaminidase had significantly increased, suggesting that the proteinuria and hematuria were not caused by glomeruli but were related to renal tubular injury. Moreover, the patient suffered from prolonged hyperchloremic acidosis and electrolyte disorders, including low potassium, sodium, and phosphorus levels after admission. These findings are consistent with the clinical manifestations of Fanconi syndrome. According to the Naranjo scale (9), the correlation between DFR and renal tubular damage scored 7 points (highly likely), suggesting that the drug was closely related to refractory acidosis and electrolyte imbalance. Bird et al. found that the DFR dose was associated with the occurrence of acute kidney injury. With each 5 mg/kg·day increase in the DFR dose, the acute kidney injury risk in patients significantly increased. When serum ferritin was lower than 1,000 μg/L, high-dose DFR [>30 mg/(kg·day)] increased the acute kidney injury risk (10). Dee et al. found that the cumulative incidence of renal tubular dysfunction was approximately 11% in patients receiving DFR de-iron therapy for two months. However, the incidence was approximately 50% and 90% in patients treated for 12–13 months and six years, respectively. A possible mechanism is that DFR causes increased serum Cr by affecting hemodynamic changes, causing an increased blood concentration of de-iron agents and iron depletion of renal cells, resulting in renal injury (11). In this case, the patient weighed 22 kg and had been on DFR >30 mg/(kg·day) for over 12 months. According to the results of Bird and Dee et al., the estimated incidence of renal tubular dysfunction in this case is at least over 50%, suggesting that DFR may be the main cause of Fanconi syndrome in this patient. However, the results of Taher et al. contradicted this case, as they found that increasing the DFR dose to >30 mg/kg·day could effectively reduce serum ferritin levels without increasing the adverse reaction incidence or worsening renal or hepatic function (12).

Few reports exist describing the association between hyperammonemia and Fanconi syndrome. Adult studies have found that renal injury can be a cause and a consequence of the pathological process that increases blood ammonia levels. In healthy patients, over 20% of the ammonia produced daily is excreted by normally functioning kidneys. In renal insufficiency cases, a progressive increase in blood ammonia levels can inhibit the excretory capacity of the renal tubules, causing hyperammonemia (13).

The patient in this report presented with hyperammonemia and Fanconi syndrome. While hyperammonemia is not uncommon in clinical practice, it has not yet been reported along with Fanconi syndrome. This report provides clinical ideas for pediatricians in clinical practice. When treating children with thalassemia using DFR, drug-induced hyperammonemia with Fanconi syndrome should be considered with an increase in blood ammonia, renal glycosuria, refractory acidosis, and electrolyte imbalance. Early detection of renal injury caused by Fanconi syndrome is essential as it is reversible through dose reduction or treatment cessation; however, failure to identify and treat the injury early may lead to serious side effects, disability, and other sequelae. Therefore, for effective management and long-term follow-up of chronic diseases in children, pediatricians must master standardized treatments and the adverse reactions of various drugs. When symptoms are difficult to explain clinically, it is necessary to trace the source and adjust the treatment plan to maximize improving patient prognoses.

## Data Availability

The original contributions presented in the study are included in the article/Supplementary Material, further inquiries can be directed to the corresponding author.
